# The Hospital Saturday Fund

**Published:** 1907-10-19

**Authors:** W. P. Treloar

**Affiliations:** Lord Mayor.


### Editor's Letter-Box.

[Our Correspondents are reminded that prolixity is a great bar to publication, and that brevity of style and conciseness of statemen greatly facilitate early insertion.]

### THE HOSPITAL SATURDAY FUND.

The Lord Mayor writes under date October 10, 1907:—

Will you permit me, as President of the Hospital Saturday Fund, to appeal for support on Hospital Saturday? Since July 1897, there has been no street collection, and the Fund lost a forcible advertisement. But the policy then adopted has proved wise, and the income of the Fund has steadily increased, until last year it was £26,460, which is £6,322 in excess of what was collected in 1897.

The steady progress made by the Fund has been the result, in a great measure, of the more general adoption of the penny-a-week and other periodical collections in the workshops and business houses of London. But a considerable sum has been collected in a quiet way on Hospital Saturday since the annual street collection was discontinued by tradespeople and others by means of boxes or sheets supplied for the purpose. As the result of last year's collection £23,898 was distributed among 205 institutions.

One feature of the work I strongly commend, namely the inculcation of the principle of self-help among the working classes. The majority of the 1,535 convalescents sent to homes paid about half the cost of their maintenance; and 5,231 surgical appliances were supplied, the recipients paying half the cost of their appliances, generally on the instalment system.

Another feature deserving of the highest commendation is the provision of suitable accommodation at sanatoria for tuberculous patients. In addition to utilising the accommodation at the consumptive hospitals, twenty beds are endowed at certain institutions, and are kept constantly occupied by suitable patients, who pay a portion of their cost of maintenance.

The Association also organises classes for teaching the principles of first-aid, and provides ambulance boxes containing the requisites for rendering first-aid for the use of workmen in factories and workshops.

All this work is carried on year by year by an army of voluntary workers, who deserve every consideration and support. I therefore appeal with confidence to the public to join in the special efforts being made through the metropolis during this week and culminating on Hospital Saturday, October 12, and shall be pleased to receive donations at the Mansion House, or they may be sent to the hon. treasurer of the Fund, Mr. H. J. Hamilton-Hoare, 54 Gray's Inn Road, W.C.—I am, Sir,

Yours faithfully,
W. P. Treloar, Lord Mayor.

THE HOSPITAL *October* 19, 1907.

